# In Vivo Assessment of Stem Cells for Treating Neurodegenerative Disease: Current Approaches and Future Prospects

**DOI:** 10.1155/2017/9751583

**Published:** 2017-02-23

**Authors:** Byeong-Wook Song

**Affiliations:** ^1^EIT/LOFUS R&D Center, Institute for Integrative Medicine, College of Medicine, Catholic Kwandong University, Gangneung-si, Gangwon-do 25601, Republic of Korea; ^2^Catholic Kwandong University International St. Mary's Hospital, 25 Simgok-Ro, 100 Beon-Gil, Seo-Gu, Incheon 22711, Republic of Korea

## Abstract

In recent years, stem cell-related therapies have been widely applied for treating neurodegenerative disease. Despite their potential, stem cell tracking and imaging techniques for the evaluation of in vivo proof-of-concept (PoC) therapies have not been sufficiently represented in the research area. This review summarizes the recent approaches that have been used for tracking and imaging engrafted stem cells in vivo. Furthermore, we introduce tissue clearing technology that can be applied to develop three-dimensional in vivo experiments. Monitoring stem cell survival and migration and graft-host relationships is a useful strategy to evaluate the therapeutic efficacy of regenerative medicine approaches in neurodegenerative disease.

## 1. Introduction

Stem cell therapy is an emerging strategy to directly restore neural function and synaptic circuit in neurodegenerative diseases such as Parkinson's disease (PD), Alzheimer's disease (AD), and Huntington's disease (HD) [1]. Many progressive therapies have been developed to reduce neuronal loss and neurological impairment through cellular replacement and other cell-based treatments, including cell culture media and extracellular vesicles [2–4]. Although stem cell research has been undertaken in a disease-specific manner, there are few currently available treatments that have applications in clinical reality. The key difficulty in achieving clinical success with stem cell therapy is the precise evaluation of the status of the transplanted cells and the condition of graft-host cells in vivo. There is an urgent need for improvement and development of noninvasive technologies for assessing the efficacy of proof-of-concept (PoC) therapy in vivo [5]. Here we review representative reports in terms of in vivo stem cell PoC therapy. In detail, we explain cell survival, therapeutic efficacy, and functional mechanisms after stem cell transplantation. We highlight the application of emerging technologies, which effectively validate in vivo PoC therapies for treating neurodegenerative diseases.

## 2. Stem Cell Tracking

In damaged brain tissue at the site of neurodegenerative disease, molecular mechanisms, synapses, neuronal subpopulations, and neural networks can be altered compared to those in normal brain tissue [6]. There are a number of major issues in carrying out assessment of PoC stem cell transplantation in vivo in these cases. First, it is necessary to analyze the efficiency and efficacy of adherent or surviving cells as compared to those of the grafted cells. Cerri et al. carried out intracarotid infusion of mesenchymal stem cells (MSCs) in a 6-hydroxydopamine (6-OHDA) rat model of PD. After a transient blood-brain barrier (BBB) opening with mannitol, MSC distribution was quantitatively analyzed 7 and 28 days after infusion. To analyze the pattern of cell distribution, colabeling with CellVue NIR815 and PKH26 differentiated infused MSCs preferentially localized in both contralateral and ipsilateral hemispheres. MSCs were more than 9.9% alive in the striatum and substantia nigra pars compacta (SNc) compared to other brain areas at 28 days [7]. Second, specific migration of transplanted cells in situ is important at the site of injury. Anderson and Caldwell reported extensive migration of human neural progenitor cells (HNPCs) when grafted into the subthalamic nucleus. Using a human-specific nuclear marker (hNuc) and a doublecortin (DCX) antibody associated with neuroblast migration, researchers could confirm that HNPCs remain in an immature state [8]. Third, it is important to determine how engrafted cells function. Gubert et al. investigated the fate of transplanted bone-marrow mononuclear cells (BMMCs) in a mouse model of amyotrophic lateral sclerosis (ALS) (SOD1^G93A^ mice). Using the fluorescent cell marker CellTrace™ and superparamagnetic iron nanoparticles (SPION) FeraTrack™, researchers verified that BMMCs interact with microglial phagocyte profiles in the lumbar spinal cord. BMMC therapy resulted in a mild temporary effect in the presymptomatic phase of ALS [9]. Finally, mapping of cell spatial distribution and connectivity in the graft-host area is important, because it allows for specific analysis of micro-environmental change brought about by stem cell therapy. Grealish et al. used a modified rabies virus to trace afferent and efferent connectivity of engrafted human embryonic stem cell- (hESC-) derived dopaminergic neurons. They found that transplanted dopaminergic neurons were integrated into the host neural circuitry in a rat model of PD [10]. This study demonstrates that special image tracing technology can convey intrastriatal graft-host connectivity in stem cell-based therapy and can easily be applied to analyze different neuronal subtypes.

To solve these problems for the assessment of in vivo PoC stem cell therapy, many histological and imaging techniques have been developed. Specific proteins, DNA/RNA-conjugated fluorescent dyes, and viral/nonviral constructs have been used to label stem cells before therapy and then used for visualization and tracking of implanted cells in vivo, using imaging modalities including magnetic resonance imaging (MRI), computed tomography (CT), positron emission tomography (PET), and near-infrared (NIR) fluorophores. Recently developed tracking and imaging techniques provide more detailed analysis of cellular phenotypes and functional analysis in stem cell therapy.

### 2.1. Cell Labeling

#### 2.1.1. Histochemical Analysis

In general, thymidine analogues have been used to detect proliferative stem cells. The thymidine analogue is a synthetic nucleoside that becomes incorporated into newly dividing DNA. Cells are treated with thymidine analogues such as bromodeoxyuridine (BrdU) to directly track transplanted stem cells. In traumatic brain injury (TBI) known as delayed-onset neurodegenerative syndrome, MSCs cultured with neurotrophic factors, including brain-derived neurotrophic factor (BDNF) and nerve growth factor (NGF), were labeled with BrdU. Eight days after TBI, these cells were found in the injected and injured sites [11]. In another study, the sphere derived from human neural stem/progenitor cells was labeled with a pulse of 10 *μ*M BruU before transplantation. After 2 or 6 weeks following implantation, engrafted spheres were found in the perilesional zone, hippocampus, corpus callosum, and ipsilateral subependymal zone of rat brains. Furthermore, at 6 weeks, stem cells had migrated and differentiated in the contralateral cortex [12]. Wennersten et al. used intraperitoneally applied BrdU for detecting endogenous stem cells or tracing cell lineage in newborns. At 2 and 4 weeks after transplantation, the number of BrdU-positive cells had increased in the hippocampus of human MSC-injected animals compared to that in AD animals, which were at normal levels. In the dentate gyrus of brains, the number of BrdU-positive cells stained with Hud (ELAV-like protein 4, ELAVL4) were also considerably elevated in the MSC-treated group (73.3% of BrdU-positive cells) compared to that in normal mice (45%). This suggests that BrdU- or BrdU/HuD-staining is a meaningful method for the detection and assessment of endogenous adult neurogenesis after MSC administration for AD treatment [13].

Using a Huntington's disease model induced by unilateral intrastriatal administration of quinolinic acid, rats received human immortalized neural stem-like cells (NSCs) infected with vector encoding *β*-galactosidase (*lac* Z). The reporter gene fuses with an intracellular promoter, and its activity is approximately the same as the steady-state mRNA level. Transplanted cells were labeled with X-gal to identify the lac Z-stained region, and migrated NSCs were found in the striatum of rats at 2 weeks after an intravenous injection of cells [14].

For long-term tracking of injected stem cells, researchers have applied fluorescent in situ hybridization (FISH). In an adult female intracerebral hemorrhage model, male MSCs were injected into the striatum, and then FISH was carried out with double staining of the chromosomal Y-linked SrY-gene and neuronal nuclei (NeuN) protein or gliofibrillary acidic protein (GFAP). After 2 months, histological analysis of graft-host integration and phenotypic expression of GFAP and NeuN was carried out [15]. In severe TBI, Sry-positive MSCs were identified by FISH analysis of colabeled with NeuN or GFAP [16].

#### 2.1.2. Fluorescent Assay

Fluorescent labels have been used to analyze the efficacy of stem cell therapy in many cases because of their ease of handling and ability to penetrate cells. Their use can be divided into three categories, according to technique and characteristics. The first of these involves applying a membrane dye that stains the cell surface, including lipophilic compounds [17]. Mouse ESCs labeled with a yellow-orange fluorescent dye with long aliphatic tails (PKH26, Sigma-Aldrich) were transplanted in the striatum in 6-OHDA-lesioned rats. At 4 weeks, ESCs were directly visualized with PKH26 costained tyrosine-hydroxylase (TH), not tumor growth [18]. Sadan et al. confirmed that, in the quinolinic acid-induced striatal lesion model for HD, MSCs secreting neurotrophic factors had migrated to adjacent sections of the risk area, by staining injected cells with PKH26 and host macrophages with macrophage marker ED1 (CD68). About 23.07 ± 7.91% of the total surviving injected cells were phagocytosed by host macrophage at site [19]. In another study, the Vybrant® series (DiO, DiI, and DiD, ThermoFisher Scientific) lipophilic carbocyanine-based dyes were used to track stem cells. With 2 *μ*g/mL of CM-DiI, MSCs were labeled and transplanted into the right cerebellar hemisphere (folia VI). After 6 weeks, DiI-labeled MSCs were found surviving and migrating in a deep layer of the cerebellar cortex [20]. Lee et al. transplanted adipose-derived stem cells (ASCs) labeled with Vybrant DiO and costained with specific antibodies including those against BDNF, calbindin, *γ*-amino butyric acid (GABA), and glutamic acid decarboxylase (GAD). In addition, they infected ASCs with virus carrying green fluorescent protein (GFP) and then implanted these in a HD model. Through these processes, the level of ASCs was doubly verified in vivo [21].

GFP-producing animals are a fundamental cell tracking tool. Various stem cells harvested from GFP-transgenic animals have been used to study neurodegenerative disease. In 2015, Hoban et al. reported functional improvement of glial cell line-derived neurotrophic factor (GDNF) induced MSCs in the lipopolysaccharide (LPS) inflammatory model of PD. Twenty-one days after transplantation, only 40% of transplanted MSCs had survived [22]. GFP-producing MSCs, macrophages, and microglia were delivered to the brain of PD and AD model mice by intranasal application. Seven days after application, labeled MSCs were found in the olfactory bulb and brain stem of (Thy1)-h[A30P] *α*S and APP/PS1 transgenic mice [23].

Sibov et al. demonstrated multimodal activity of iron oxide nanoparticles linked with Rhodamine-B (MION-Rh, BioPAL Inc.) in umbilical cord blood (UC-) MSCs therapy. They found that UC-MSCs labeled with MION-Rh were detectable by MRI in an animal model of PD. These cells slowly migrated from the medial forebrain bundle to the substantia nigra, where they were detected by T2 MRI as hypointense spots [24].

### 2.2. Imaging Technology

Researchers need to be able to identify stem cells in vivo at the cellular and molecular level for tracking and assessing therapeutic efficacy. Noninvasive imaging techniques enable researchers to understand the exact in vivo stem cell therapy PoC in neurodegenerative disease. Here we review the specific imaging techniques and the future goals for clinical study.

#### 2.2.1. MRI and PET

Among noninvasive techniques, MRI provides the highest spatial resolution for acquiring cellular and molecular images. Modo et al. performed MRI to track engrafted stem cells in an animal study of brain disease for the first time [25]. Owing to significant T2 effect, iron nanoparticles have been used in stem cell tracking instead of gadolinium-based particles [26]. HNPCs overexpressing ferritin, an iron storage protein, were found in the striatum of rat brains by MRI. Furthermore, hypointensity was increased in SPION-labeled HNPCs [27]. In studying the migratory process of neurotrophic factors-secreting MSCs, MRI was also used for high-resolution 2D and 3D imaging in HD [19]. In TBI therapy, HNPCs treated with ultrasmall SPION (USPION) and/or Molday ION Rhodamine-B (MIRB™) had been identified with increased homing and retention, using 0.6T MRI [28]. Furthermore, MRI has been used to assess a diagnostic strategy by finding common biomarkers of AD after stem cell transplantation [29].

PET in combination with MRI can provide information on importance of in vivo PoC parameters, including optimal cell dose, cell tracking, graft volume, and functional assessment [30]. To define a preclinical assessment, Grealish et al. reported long-term survival of hESC-derived midbrain dopamine neurons in a rat PD model. Using MRI and PET imaging techniques, the striatal dopaminergic neurotransmission (^18^F-fallypride as D_2_/D_3_-receptor occupancy) and the dopamine transporter (DAT, ^18^F-LBT999 as a tracer) were assessed [31].

#### 2.2.2. SPECT and NIR

SPECT is a nuclear tomographic imaging technique that uses a gamma ray camera. In hemiparkinsonian rhesus monkeys, dopaminergic function was measured by DAT activity in a MSC-derived dopaminergic neuron-like cell-transplanted brain [32]. Furthermore, it is suggested that [^123^I]-2*β*-carbomethoxy-3*β*-(4-fluorophenyl)-N-(3-iodo-E-allyl)nortropane ([^123^I]altropane) can be used to identify DAT. This suggests that SPECT-[^123^I]altropane is a specific marker to measure dopaminergic content for functional verification [33].

Near-infrared light (NIR) imaging has the advantage of noninvasive detection, while being capable of penetrating deep into tissues. Human MSCs were detected and quantified using CellVue® NIR815 and NIR scanning to render contour, reconstruction, and coronal section at the cell-engrafted site in 3D. Finally, NIR815-hMSC localization was measured in vivo over time [34].

## 3. Future Directions

An important development in assessing stem cell therapy with brain imaging is the ability to measure the relationship and connectivity between graft and host cells in 3D. The CLARITY (Clear, Lipid-exchanged, Acrylamide-hybridized Rigid, Imaging/immunostaining compatible, Tissue hYdrogel) method is the best currently available tool for analyzing graft-host relationships between stem cells and diseased tissue. This technique was first developed by Karl Deisseroth from Stanford University [35]. For building hydrogel-tissue hybridization structures, a hydrogel monomer is infused into whole brain tissue. SDS micelle then captures lipids in the tissue, which leads to tissue transparency during electrophoresis. Stem cells are labeled with specific dyes before the animals are killed and the clear tissue is then immunostained. Data is acquired by confocal, two-photon, or light-sheet microscopy and analyzed by 3D rendering software. Other tissue clearing techniques are ACT-PRESTO (active clarity technique-pressure related efficient and stable transfer of macromolecules into organs) [36], PACT (passive clarity technique) [37], and uDISCO (ultimate 3D imaging of solvent-cleared organs) [38].

In this review, conventional stem cell tracking and assessing techniques have been discussed, including noninvasive imaging methods. This review has demonstrated that these techniques can help the assessment of in vivo PoC stem cell therapy and thus serve as the basis for clinical monitoring of neurodegenerative disease.
